# Age but not sex modifies lymphoid immune responses in murine sepsis

**DOI:** 10.21203/rs.3.rs-9751570/v1

**Published:** 2026-06-25

**Authors:** Dayuan Wang, Christine Rodhouse, Hongru Tang, Miguel Hernández-Ríos, Xuanxuan Yu, Ruoxuan Wu, Whitman Wiggins, Marvin L. Dirain, Ricardo Ungaro, Dina C. Nacionales, Lyle L. Moldawer, Shawn Larson, Michael Kladde, Clayton Mathews, Paramita Chakrabarty, Gemma Casadesus, Jaimar C. Rincon, Larissa Langhi Prata, Feifei Xiao, Maigan A. Brusko, Robert Maile, Philip A. Efron, Guoshuai Cai

**Affiliations:** University of Florida; University of Florida; University of Florida; University of Florida; University of Florida; University of Florida; University of Florida; University of Florida; University of Florida; University of Florida; University of Florida; University of Florida; University of Florida; University of Florida; University of Florida; University of Florida; University of Florida; Cedars-Sinai Medical Center; University of Florida; University of Florida; University of Florida; University of Florida; University of Florida

**Keywords:** single-cell RNA sequencing, aging, immunosenescence, T and B cells

## Abstract

Sepsis disproportionately affects the elderly, and the cellular mechanisms driving age- and sex-dependent lymphoid immune remodeling remain poorly defined. In this work, we mapped the splenic lymphoid transcriptional landscape of young and older adult, male and female mice after sepsis by single-cell RNA sequencing. While both sexual and age dimorphism shaped the baseline lymphocyte composition, the transcriptional reprogramming induced by sepsis was significantly influenced only by age. Sepsis induced a proportional reduction in lymphocytes across age and sex groups; however, aging modified the pattern of lymphocyte reconstitution. Following sepsis, older adult mice displayed an enhanced B cell maturation compared to young mice. Moreover, across all major lymphocyte subtypes, older adult mice demonstrated transcriptionally suppressed metabolic pathways at baseline that shifted to exaggerated activation after sepsis. Furthermore, intercellular communication analysis from antigen-presenting cells to T cells revealed broadly age-dependent activation of co-stimulatory and antigen-presentation pathways after sepsis. Age and sepsis also widely reshaped the druggable landscape in lymphocytes, revealing a distinct predicted drug response profile in older adult mice after sepsis. These data suggest that aging reshapes the lymphoid baseline and the subsequent septic response in ways that may contribute to the poorer outcomes observed in older hosts. Notably, under these conditions, we did not detect significant sexual dimorphism. This lymphoid-specific age-driven transcriptional override highlights specific metabolic and signaling checkpoints as potential targets for precision immunotherapy in older sepsis.

## Introduction

Sepsis, defined as organ failure/insufficiency caused by a dysregulated host response to infection^1^, is a devastating condition due to its high incidence, costly hospital care, and long-term disability^2^. This places a massive economic and societal burden on patients, families, and healthcare systems^3^. Although in-hospital mortality has decreased due to efforts such as the Surviving Sepsis Campaign^4^, the incidence of sepsis continues to rise and long-term outcomes for patients remain poor^5–7^. To date, despite decades of research, little progress has been made in identifying immunomodulators to improve patient outcomes^8–10^.

A central driver of poor prognosis for sepsis is the profound and persistent dysregulation of lymphocytes. It has been well established that the loss of lymphocytes as well as their suboptimal function after sepsis contribute to the poor immune state of the host^11–15^. This is due to multiple factors, including but not limited to lymphocyte loss (e.g. apoptosis), a shift away from lymphopoiesis in progenitor cells, and dysfunction of the lymphocytes present in the infected individual^16,17^. These same changes are also observed during chronological aging, as part of natural age-related immune decline called immunosenescence. It is influenced (or driven) by chronic low-grade systemic inflammation (inflammaging), and results in dysregulation of immune surveillance^18,19^. Previously, most sepsis research revolved around CD4^+^ T lymphocytes^20^; however, more recently, interest has emerged regarding other lymphocyte subsets (e.g. CD8^+^ T lymphocytes, B cells, Natural Killer cells (NKs))^20–23^. These other cells, as well as their potential interaction with antigen-presenting cells (APCs), remain understudied in sepsis. Understanding how these populations interact is critical for reversing the persistent inflammation, immunosuppression, and catabolism syndrome seen in some sepsis survivors^24^, and especially how the aged immune system influences these interactions, as age affects the severity of sepsis outcomes.

Another key aspect of the lack of progress in sepsis therapeutics is the failure to incorporate the principles of personalized medicine to approach septic patients^6^. Although IL-7 and IFN-γ hold promise in improving host immune function after severe infection^25,26^, the “silver bullet” concept of one drug working for all individuals is highly unlikely to work in diseases of systemic inflammation and shock^24^. A growing body of evidence indicates that the most rudimentary differences in mammals, such as age and sex, can fundamentally influence the immune system at baseline and in response to sepsis^27–31^. In murine sepsis models, older age is similarly associated with worse outcomes and higher mortality^32,33^. Consequently, a better understanding of the differences in lymphocyte alterations after sepsis as a function of age and sex will allow investigators to take more nuanced approaches to restore immune homeostasis in the sepsis survivor^30^.

Single-cell RNA-seq (scRNA-seq) allows the simultaneous analysis of the entire transcriptome of individual cells, enabling the mapping of a single-cell gene expression atlas. In this study, we mapped the splenic lymphoid landscape using a murine model of sepsis that better recapitulates the human condition than historical models^34,35^. We studied the effect of age and sex in lymphocyte dysregulation after sepsis and found a significant modification effect of age but not sex on cell compositions, cell type-specific transcriptomic profiles, cell differentiation, intercellular communication networks and drug target landscapes. These findings could highlight which cell subsets and pathways are key to age-dependent lymphoid dysfunction. Elucidating the mechanisms underlying worse outcomes in older adults will aid in directing therapeutic approaches to specific patient age groups.

## Results

### A single-cell atlas of septic mouse spleen reveals diverse immune populations.

To define the splenic immune landscape in naive and septic mice, we profiled splenocytes from 57 total mice (N = 28 naive, 29 septic) using scRNA-seq. The cohort was balanced across experimental conditions, including age (older adult [18–24 months] vs. young [3–4 months]), sex (female vs. male), and disease status (CLP + DCS 7d vs. naive) (**Supplementary Table 1**). Consistent with prior characterization of this model^29,33^, seven-day septic mortality was higher in older adult than young adult mice (log-rank test, p = 0.025). Following rigorous quality control and batch integration (see Methods), we obtained a high-quality single cell-transcriptomic dataset of 828,744 total cells. The dataset was robust at both the sample and cell level, with a median of 13,527 cells captured per sample and a median of 1,024 genes and 1,883 UMIs per cell.

**Figure 1:**
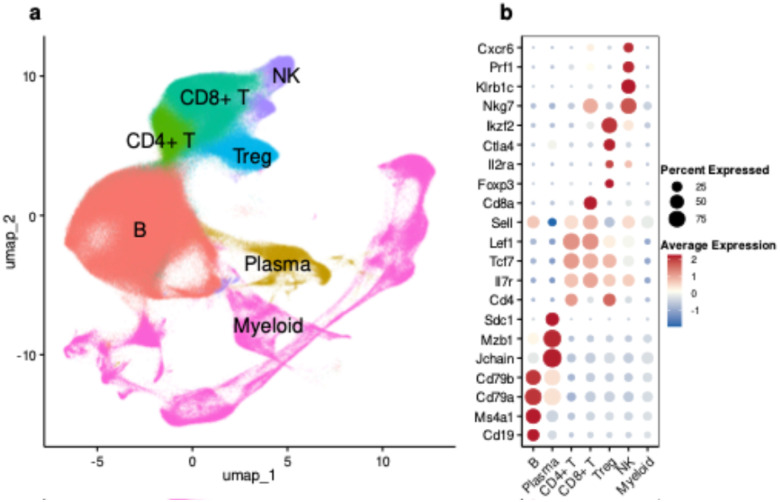
Single-cell atlas of splenic immune cells. (a) Uniform Manifold Approximation and Projection (UMAP) visualization of splenocytes identifying major immune cell populations. The lymphoid compartment resolves into B cells, plasma cells, CD4+ T cells, CD8+ T cells, CD4+CD25+ Regulatory T (Treg) cells, and Natural Killer (NK) cells, alongside a broader myeloid population. (b) Dot plot characterizing the expression of canonical marker genes used for cell type annotation. Dot size represents the percentage of cells expressing the marker, while color intensity indicates the average scaled expression level. Key markers include B cells (Cd19, Ms4a1, Cd79a, Cd79b), plasma cells (Jchain, Mzb1, Sdc1), T cells (Il7r, Tcf7, Lef1, Sell), CD4+ T cells (Cd4), CD8+ T cells (Cd8a), Treg cells (Foxp3, Il2ra, Ctla4, Ikzf2), and NK cells (Nkg7, Klrb1c, Prf1, Cxcr6).

Unsupervised clustering and UMAP visualization resolved distinct immune lineages, capturing both the lymphoid and myeloid compartments (Fig. 1a). Cell identities were assigned based on the expression of canonical markers (Fig. 1b). The lymphoid compartment consisted of B cells (*Cd19*, *Ms4a1*, *Cd79a*, *Cd79b*), plasma cells (*Jchain*, *Mzb1*, *Sdc1*), natural killer (NK) cells (*Nkg7*, *Klrb1c*, *Prf1*, *Cxcr6*), and T cells (*Il7r*, *Tcf7*, *Lef1, Sell*). This T cell population was further resolved into CD4^+^ T cells (*Cd4*), CD8^+^ T cells (*Cd8a*), and Treg cells (*Foxp3*, *Il2ra*, *Ctla4*, *Ikzf2*). Parallel analysis of the myeloid compartment identified macrophages (*C1qa*, *Adgre1*), dendritic cells (DCs) (*H2-Aa*, *Itgax*), polymorphonuclear cells (PMNs) (*S100a8*, *S100a9*, *S100a11*), monocytes (*F13a*, *Ms4a6c*, *Ly6c2*), myeloid-derived suppressor cells (MDSCs) (*Cers6*, *Igf1r*, *Itgam*)^36^, and common myeloid progenitors (CMPs) (*Tyms*, *Rrm2*), as shown in **Supplementary Fig. 1**.

### Sepsis induces age-dependent remodeling of lymphocyte populations.

**Figure 2:**
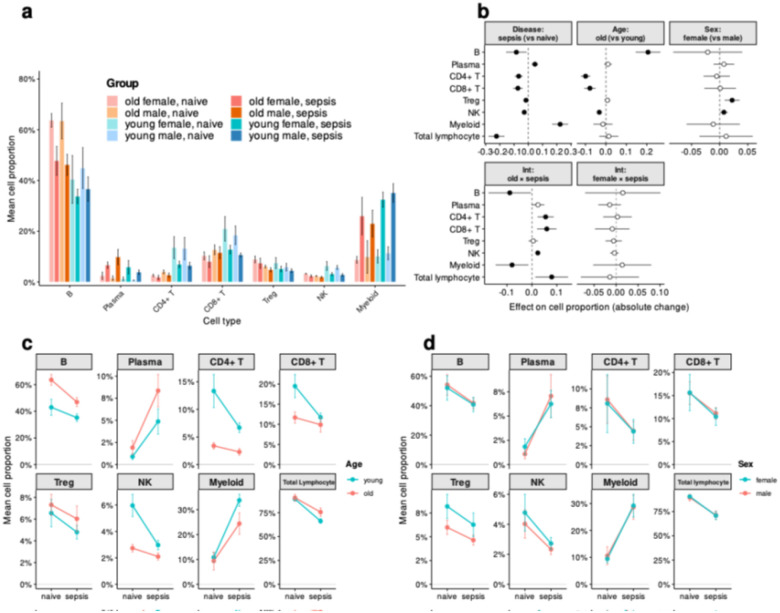
Lymphocyte composition is shaped by age-dependent responses to sepsis. (a) Stacked bar plots showing the mean proportion of lymphocyte and myeloid subsets across all experimental groups (naive/sepsis, young/old, male/female). (b) Forest plots displaying the linear model estimates (β) for the main effects (sepsis, age, sex) as well as interaction terms. Points represent the coefficient estimate and error bars denote 95% Wald confidence intervals. Filled circles indicate statistically significant effects (p<0.05). (c) Interaction line plots illustrating the effect of sepsis across age groups. The plots show clearly non-parallel lines, consistent with a significant interaction effect. (d) Interaction line plots illustrating the effect of sepsis across sex groups. The plots show nearly parallel lines, indicating minimal interaction between sex and sepsis.

With the splenic cell atlas, we next evaluated how cell type proportions were altered by sepsis, age, and sex, with interaction effects determined via the statistical model shown in Eq. 1. Our data revealed significant decrease in proportion of B cells (41.3% in sepsis vs. 53.3% in naive, Δ =−12%, p = 0.03), CD4^+^ T cells (4.4% in sepsis vs. 8.4% in naive, Δ =−4%, p < 0.001), CD8^+^ T cells (10.8% in sepsis vs. 15.5% in naive, Δ =−4.7%, p < 0.001), and NK cells (2.5% in sepsis vs. 4.3% in naive, Δ =−1.8%, p < 0.001). In contrast, plasma cells were significantly expanded in septic cohorts (4.7% in sepsis vs. 1.4% in naive, Δ =3.3%, p < 0.001) (Fig. 2b, condition: sepsis (vs. naive) panel). Compared to young adult mice, older adult mice had significantly smaller baseline proportions of CD4^+^ T (3.4% in naive older adult vs. 13.3% in naive young, Δ =−9.9%, p < 0.001), CD8^+^ T (11.7% in naive older adult vs. 19.5% in naive young, Δ =−7.8%, p < 0.001), and NK cells (2.7% in naive older adult vs. 6.0% in naive young, Δ =−3.3%, p < 0.001), but a significantly larger B cell compartment (63.6% in naive older adult vs. 43.0% in naive young adult, Δ =20.6%, p < 0.001) (Fig. 2b, age: older adult (vs. young) panel). Sex-based differences were present but less pronounced: compared to male mice, female mice exhibited larger baseline proportions of Treg cells (8.2% in naive female vs. 6.0% in naive male, Δ =2.2%, p = 0.002) and NK cells (4.7% in naive female vs. 4.0% in naive male, Δ =0.7%, p = 0.037) but no difference in other types of lymphocytes (Fig. 2b, sex: female (vs. male) panel).

A significant age-dependent effect of sepsis was observed that, while sepsis caused overall decrease in lymphocyte proportion in both older and young adult mice, this decrease was significantly less pronounced in older adult mice (interaction *β* =−0.08, p = 0.02, Fig. 2b, c). Specifically, this age-dependent effect was characterized by the less decrease of CD4^+^ T (interaction *β* =0.055, p = 0.0015), CD8^+^ T (interaction *β* =0.060, p = 0.004), and NK cells (interaction *β* =0.024, p < 0.001) in older adults (Fig. 2b, c). In contrast, in older adult mice, the sepsis-induced B cell decrease (interaction *β* =−0.088, p = 0.046) and plasma cell expansion (interaction *β* =0.024, p = 0.067) was more pronounced. No sex-dependent effects were observed (Fig. 2b, d).

Within the lymphocytes, significant modification effect of aging was also found in the depletion of CD4^+^ T (interaction *β* =0.037, p = 0.047), NK cells (interaction *β* =0.019, p < 0.001), and an inverted direction of change in B cells (interaction *β* =−0.122, p = 0.013) (**Supplementary Fig. 2**).

### Age-dependent immune and metabolic transcriptional changes in lymphocytes after sepsis.

Among the lymphocyte cell types, sepsis was associated with expression changes in 776–5,866 genes, while aging was associated with the expression changes in 5,976–9,459 genes (Table 1). In contrast, biological sex displayed minimal impact on the transcriptional profile, with fewer than 200 differentially expressed genes (DEGs) detected in any lymphocyte population (Table 1). These findings suggested extensive transcriptional remodeling in response to both sepsis and aging but not sex across all cell types.

**Figure 3:**
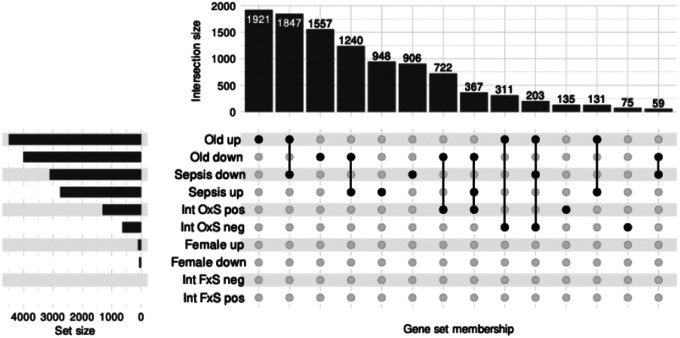
Age-dependent transcriptional dysregulation in B cells. UpSet plot showing intersections between differentially expressed gene sets derived from main effects and interaction terms of the linear model. Each intersection represents the overlap between gene sets defined by age, sepsis, and age × sepsis model terms. Horizontal bars indicate the total number of genes in each gene set, while vertical bars represent the number of genes within each intersection. Two large intersections correspond to genes elevated with age but reduced during sepsis (age up, sepsis down; 1847 genes) and genes reduced with age but increased during sepsis (age down, sepsis up; 1240 genes). Among the interaction-associated intersections, one group (367 genes) includes genes reduced at baseline in older adult mice (age down) but increased during sepsis (sepsis up) with a positive age × sepsis interaction. A second group (203 genes) includes genes elevated at baseline in older adult mice (age up) but decreased during sepsis (sepsis down) with a negative age × sepsis interaction. Only gene sets containing more than 50 genes are shown.

Interestingly, B cells exhibited a large overlap between genes affected by age and those affected by sepsis (3,863 shared genes out of 8,509 age-related DEGs and 5,866 sepsis-related DEGs). A significant inverse relationship between these factors was observed (Fisher’s exact test, p < 0.001), that 91.6% (1,615 of 1,763) of sepsis-upregulated genes were downregulated with aging, while 96.1% (2,071 of 2,154) of sepsis-downregulated genes were upregulated with aging (Table 2). Among the genes that were downregulated at baseline in older adult mice compared with young mice, 1119 out of 1312 showed significantly greater induction after sepsis (Odds ratio = 21, Chi-squared test p < 0.001); among the genes that were upregulated at baseline in older adult mice compared with young mice, 535 out of 642 exhibited significantly stronger repression after sepsis (Odds ratio = 13.5, Chi-squared test p < 0.001) (Table 2, Fig. 3).

**Figure 4:**
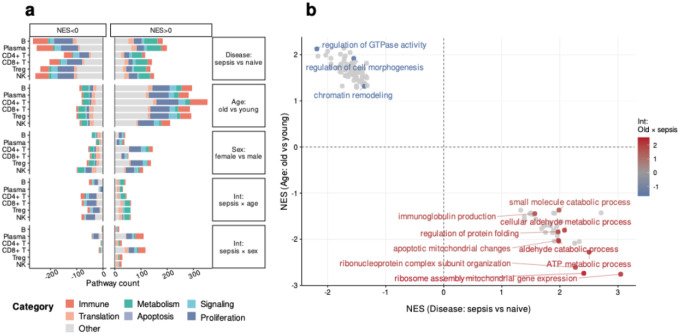
Pathway enrichment across lymphocyte subsets during sepsis and aging. **(a)** Bar plots showing the number of significantly enriched gene ontology (GO) pathways (FDR<0.05) across lymphocyte subsets. Pathways are grouped by functional category, including immune, metabolism, signaling, proliferation and development, translation, apoptosis, and other biological processes. Panels correspond to pathways associated with the sepsis main effect, age main effect, sex main effect, and interaction terms. **(b)**Scatter plots of normalized enrichment scores (NES) comparing the sepsis main effect (x-axis) and age main effect (y-axis) for enriched pathways in B cells. Each point represents an enriched GO pathway. Point color indicates the magnitude and direction of the age × sepsis interaction NES, ranging from blue (negative) to red (positive). Pathways with statistically significant interaction terms are highlighted, including immunoglobulin production. Most pathways cluster in the top-left (Sepsis ↓, Age ↑) or bottom-right (Sepsis ↑, Age ↓) quadrants, indicating an inverse relationship.

Across all lymphocyte subsets, gene set enrichment analysis revealed a consistent, age-dependent reprogramming of pathway activity after sepsis (Fig. 4). Older adult mice exhibited a baseline state characterized by reduced metabolic activity (39 upregulated vs. 55 downregulated), elevated immune (115 upregulated vs. 25 downregulated) and proliferation-related (192 upregulated vs. 26 downregulated) pathways. Conversely, sepsis was characterized by elevated metabolic pathways (110 upregulated vs. 27 downregulated) but reduced immune (41 upregulated vs. 153 downregulated) and proliferation-related (64 upregulated vs.193 downregulated) pathways. Among these pathways, metabolic pathways that were largely suppressed at baseline in older adult lymphocytes showed exaggerated induction after sepsis (38 positive vs. 10 negative interactions), whereas immune (15 positive vs. 39 negative interactions) and proliferative pathways (5 positive vs. 99 negative interactions) that were largely elevated at baseline in older adults underwent disproportionately strong inhibition in response to sepsis compared with young adults.

Taken together, these findings indicate that aging leads to a dysregulated transcriptional baseline in lymphocytes that is broadly reciprocal to the sepsis-induced transcriptional state, suggesting that the dysregulated aged baseline drives lymphocytes to exaggerated transcriptional perturbation during sepsis. These involved changes specific to cell types. In B cells, the immunoglobulin production pathway showed reduced baseline enrichment in older adult mice followed by pronounced induction after sepsis (Fig. 4b) (Interaction normalized enrichment score (NES): 0.34, q = 0.0016). The other cell type results were shown in **Supplementary Fig. 3**. T and NK cells from older adult mice showed age-dependent enrichment of pathways associated with mitochondrial apoptotic processes after sepsis, including pathways related to mitochondrial membrane permeabilization and intrinsic apoptosis.

### Age-dependent alterations in APC-to-T cell communication after sepsis.

Next, we delineated and analyzed the cell communication between the T cells and antigen-presenting cells (APCs) which included B cells, plasma cells, macrophages and dendritic cells (cDC1, cDC2 and pDC).

**Figure 5:**
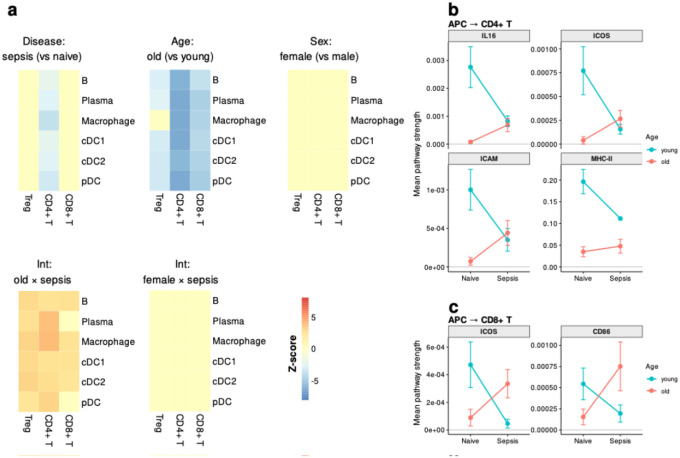
Age-dependent changes in antigen-presenting cell to T cell communication during sepsis. **(a)** Heatmaps of signed t-statistics from linear models quantifying the effects of disease (sepsis vs. naive), age (older adult vs. young), and sex (female vs. male) on aggregated cell-cell communication strength, together with the two interaction terms (age × sepsis, sex × sepsis). Rows represent antigen-presenting cell populations (B, plasma, macrophage, cDC1, cDC2, pDC) and columns represent T cell subsets (Treg, CD4^+^ T, CD8^+^ T). Reference groups for the model were naive (for sepsis), young adult (for age), and male (for sex). Positive t-statistics indicate increased communication strength relative to the reference group; negative values indicate decreased strength. For the interaction terms, positive values indicate that the sepsis-associated change is greater in older adult (or female) mice than in young adult (or male) mice. **(b)** Interaction line plots for ligand-receptor (L-R) signaling pathways from antigen-presenting cells to CD4^+^ T cells that show significant age × sepsis interaction effects, including IL16, ICOS, ICAM, and MHC-II. **(c)** Interaction line plots for L-R signaling pathways from antigen-presenting cells to CD8+ T cells, including ICOS and CD86. In (b) and (c), lines connect group means for naive and sepsis conditions within each age group (young in blue, older adult in red); points represent mean pathway strength and error bars represent standard error.

We modeled the total communications that were inferred from scRNA-seq data (**see** Methods) and evaluated the effect of sepsis, age and sex, and their interactions. Older adult mice exhibited a general weakening of overall communication from all APCs to T cell subtypes, most pronounced to CD4^+^ T cells. After sepsis, we found a reduced communication from APCs to CD4^+^ T cells but not to CD8^+^ and Treg cells (Fig. 5a). Across all the APCs, we observed a broadly attenuated age × sepsis interaction effect on APC-to-T cell communication, indicating that the sepsis-associated decline in signaling strength was less pronounced in older adult mice (i.e., less negatively affected) compared with young adults (p < 0.05). In contrast, sex was neither a significant factor in altering overall communication strength at baseline nor in modifying that after sepsis.

Several ligand-receptor signaling pathways exhibited significant age-dependent modulation in response to sepsis (Fig. 5b, c). For APC → CD4^+^ T signaling, IL16, ICOS, ICAM and MHC-II all showed lower inferred communication strength at baseline in older adult mice; after sepsis, these pathways declined further in young adults but were maintained or relatively elevated in older adults, generating a clear age × sepsis interaction (Fig. 5b). For APC → CD8^+^ T signaling, ICOS and CD86 followed the same pattern, with sepsis-induced suppression in young adults and a relative increase in older adults (Fig. 5c).

### Age-dependent B cell differentiation trajectories after sepsis.

**Figure 6:**
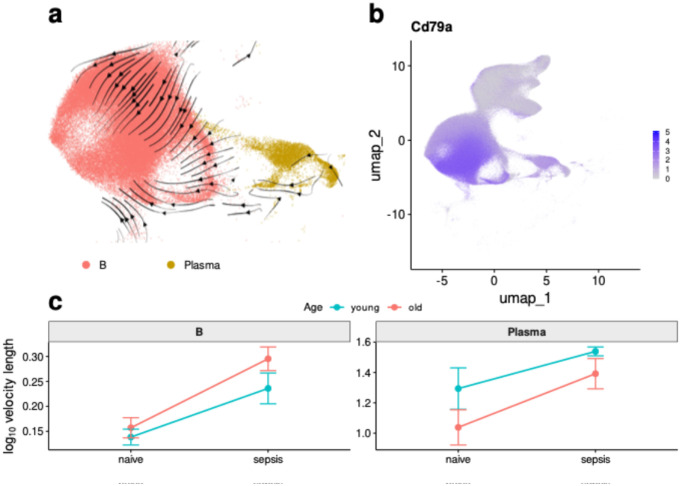
Age-dependent differences in B cell differentiation trajectories during sepsis. **(a)** RNA velocity analysis visualized on the UMAP embedding of splenic B cells and plasma cells. Streamlines (arrows) indicate the estimated future transcriptional state of individual cells, revealing a directional trajectory originating in the B cell cluster and terminating in the plasma cell cluster. **(b)** Feature plots showing expression of the canonical B cell markers *Cd79a*, confirming B cell lineage identity along the trajectory. **(c)** Interaction plots quantifying the mean RNA velocity length (log10 scale) for the age × sepsis interaction in plasma cells and B cells. Lines connect group means across naive and sepsis conditions for young and older adult mice. The non-parallel trends between age groups indicate an interaction between age and sepsis in velocity length. Error bars represent 95% confidence intervals.

To understand B cell differentiation in sepsis, we inferred the cell level RNA velocity to map the magnitude and direction of their differentiation trajectory. In sepsis, B cells exhibited velocity directed toward a transcriptional state characterized by higher expression of *Cd79a*, a marker associated with B cell maturation (Fig. 6b). Such inferred driving force from sepsis was stronger than age or sex, with a significant increase in velocity for both B cells (Fold-Change [FC] = 1.5, p < 0.05) and plasma cells (FC = 3.5, p < 0.05) (Fig. 6c). Age was associated with a significant modification effect on this sepsis-driving differentiation (FC = 1.4, p < 0.05). While the baseline differentiation state is similar, the B cell differentiation process became more activated in response to sepsis in older adult mice. This aligns with our previous finding (Fig. 2) that the sepsis-induced shift from B cells to plasma cells was significantly stronger in older adult mice. In addition, plasma cells demonstrated a significantly lower baseline velocity in older adult mice (FC = 0.4, p < 0.05, a 60% decrease), which was strongly induced by sepsis in parallel with the young adult cohort. The effect of sex, or its interaction with sepsis, were not significant for either B or plasma cells.

### Age-dependent therapeutic targets in lymphocytes.

To study the effect of age and sex on potential cell-type-specific therapeutic targets in sepsis, we mapped single-cell transcriptomic data to FDA-approved drugs via their molecular targets using Drug2cell^37^. This analysis estimates transcriptionally inferred target engagement potential.

**Figure 7:**
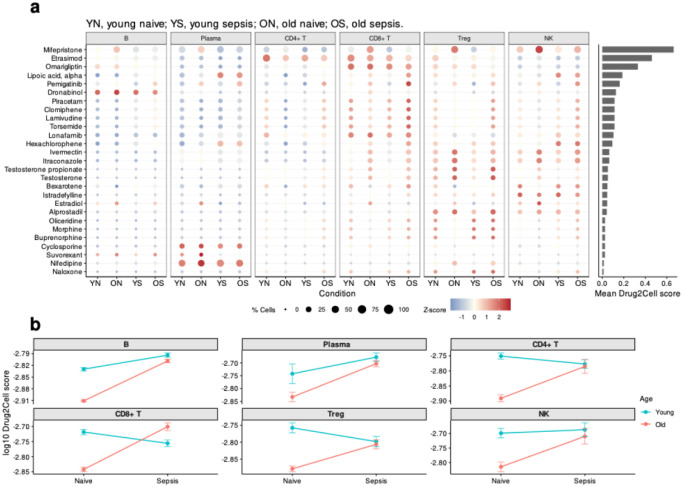
Drug2cell analysis reveals age-dependent therapeutic landscape across lymphocyte populations after sepsis. **(a)** Bubble plot summarizing inferred drug-cell targeting potential across lymphocyte subsets (B cells, plasma cells, CD4^+^ T cells, CD8^+^ T cells, Treg cells, and NK cells) stratified by age and disease status. Bubble size represents the proportion of cells expressing drug targets, and color indicates standardized drug target activity. **(b)** Interaction plots for the drug bexarotene showing mean drug target score across naive and sepsis conditions for each lymphocyte subset. Lines connect group means for young and older adult mice. The non-parallel trends between age groups indicate an age × sepsis interaction in inferred drug target activity. Points represent sample-level means and error bars indicate 95% confidence intervals.

Both sepsis and age significantly reshaped the expression of numerous drug targets across lymphocyte subsets, with 23 drugs demonstrating significant sepsis-associated shifts in predicted targeting potential (Fig. 7a **and Supplementary Fig. 4**). Among these, 16 drugs exhibited significant age × sepsis interaction effects, indicating that the transcriptional accessibility of their molecular targets differed between young and older adult mice during sepsis.

Within this interaction set, several compounds showed age-dependent shifts after sepsis (e.g., bexarotene, alprostadil, morphine, **Supplementary Fig. 4**). In all lymphocytes, bexarotene, which targets on RXR pathway demonstrated increased inferred target engagement (log2FC = 0.57, p = 0.0057) from older adult septic mice relative to young adults (Fig. 7b). These findings indicated that the underlying molecular networks engaged by these drug targets were differentially regulated by age after sepsis. Consistent with our broader transcriptomic analyses, sex had minimal impact on the inferred druggable landscape within lymphocytes, with negligible differences in drug-cell associations between male and female mice (**Supplementary Fig. 4**).

## Discussion

In this study, we performed a comprehensive single-cell transcriptomic analysis of splenic lymphocytes in a murine sepsis model to define how age and sex influence immune responses at the cellular and molecular levels. It directly assessed that difference between older and young adult septic mice which showed different seven-day mortality, consistent with the higher sepsis mortality seen in older human patients^38,39,33^. First, sepsis induced marked remodeling of the splenic lymphoid compartment, including broad lymphodepletion and plasma-cell expansion. Second, and consistent with prior studies showing profound immune remodeling in older adult sepsis patients and animal model^40,41^, age, rather than sex, was the dominant host factor modifying lymphoid immune composition, transcriptional programs, inferred intercellular communication, differentiation dynamics, and predicted druggable landscapes after sepsis (Table 3). Third, the strongest age-associated changes converged on B-cell/plasma-cell remodeling, altered inferred APC-to-T-cell signaling, and widespread reorganization of metabolic and immune pathways.

The relative absence of sexual dimorphism in lymphocytes is notable, as our prior studies in the myeloid compartment have shown significant age- and sex-dependent differences in innate immune responses to sepsis^31,39^. Together, these findings indicate that host aging, rather than biological sex, is the principal determinant of lymphoid transcriptional remodeling during sepsis. This should be interpreted within the context of the present experimental design rather than as evidence that sex is irrelevant to immunity against sepsis. In lymphoid compartment, sex had only modest effects on lymphocyte composition, differential gene expression, inferred communication networks, RNA velocity, and predicted drug-target activity, whereas age showed broad and consistent effects across all of these domains. This contrasts with our prior work in myeloid cells^31^, in which both age and sex significantly shaped the septic response, and suggests that host-factor effects may be compartment-specific.

While sex effects in lymphocytes may be subtler, tissue-specific, or temporally restricted, our central observation in this study was age-dependent remodeling of the lymphoid compartment before and after sepsis. Although sepsis caused lymphodepletion across all groups, older adult mice began from a distinct baseline state characterized by lower proportions of CD4^+^ T cells, CD8^+^ T cells, and NK cells, together with an expanded B-cell compartment. The immune cell profiling we see in aging highly correlates with the literature^42,43^. After sepsis, this baseline difference was not simply preserved; rather, the pattern of remodeling itself differed by age. Older adult mice showed relatively less proportional depletion of T- and NK-cell populations, but a more pronounced shift within the B-cell compartment, with greater B-cell loss and plasma-cell expansion^44^. These findings suggest that aging does not merely attenuate or amplify a uniform septic response, but instead redirects lymphoid remodeling toward a different cellular endpoint, particularly within humoral immune populations.

At the transcriptional level, the relationship between aging and sepsis was notable for its reciprocity. Our findings revealed extensive overlap between age-associated and sepsis-associated gene expression changes, particularly within B cells. However, the majority of genes upregulated after sepsis were downregulated at baseline in older adult mice, and *vice versa*. This reciprocal pattern suggests that aging establishes a dysregulated baseline transcriptional state, which likely leads to exaggerated transcriptional responses upon sepsis. This was reflected in the tendency for upregulated genes in older adult mice to be more strongly repressed after sepsis, and vice versa. Consistent with this interpretation, pathway enrichment analysis revealed that lymphocytes from older adult mice exhibited compromised baseline metabolic activity alongside activated immune and proliferative pathways. After sepsis, these pathways became strongly regulated in the opposite direction. These transcriptional responses were accompanied by age-dependent enrichment of mitochondrial apoptotic and stress-response pathways in T and NK cells, suggesting that exaggerated activation in the context of impaired baseline metabolic readiness may promote functional exhaustion rather than effective immunity. Prominently in B cells, immunoglobulin production pathways showed marked age-dependent upregulation^45^. In older adult mice, research has demonstrated that B cells often produce more autoantibodies and lower-affinity antibodies^46,47^; B cells show altered metabolic activity that directly primes T cells toward a more inflammatory and dysfunctional phenotype through glycolytic-generated lactate as a metabolic mediator^48^. Our findings further support this metabolic-immune axis with aging, as the observed rapid shift from a suppressed baseline to an ‘exaggerated’ transcriptional state in the lymphoid compartment of older adults likely reflects a maladaptive transcriptional attempt. This sepsis-induced metabolic and transcriptional hyperactivity, occurring within a pre-existing landscape of immunosenescence, likely contributes to the poor clinical outcomes observed in older adults.

All these observations may link to the age-dependent differentiation of B cells after sepsis. While sepsis was associated with accelerated B cell differentiation toward maturation, this acceleration appeared more pronounced in older adult mice. This aligns with the age-dependent depletion of B cells and expansion of plasma cells after sepsis that was observed in this study^41,49^.

Dysregulated antigen-presenting cells including the B cells, can alter T cell activation and differentiation through aberrant antigen presentation, co-stimulation, and cytokine signaling. Our data show that older adult mice exhibited reduced baseline readiness across several APC-to-T cell signaling pathways that are central to antigen presentation, T cell co-stimulation, and adhesion. For APC → CD4^+^ T signaling, the affected pathways included MHC-II (the principal antigen-presentation route to CD4^+^ T cells), IL16 (a CD4 ligand that recruits and activates CD4^+^ T cells), ICOS (a CD28-family co-stimulatory pathway), and ICAM (LFA-1 ligand mediating T cell adhesion and immune-synapse stabilization). For APC → CD8^+^ T signaling, ICOS and CD86 (a B7-family co-stimulatory ligand for CD28/CTLA-4) were the dominant age-modulated pathways. In each case, baseline signaling was lower in older adult mice, and the sepsis response diverged between age groups, with young adults losing signaling and older adults either maintaining or transiently regaining it^20,50–52^.

Together, these findings indicate that impaired baseline metabolic readiness in aging lymphocytes may constrain effective immune function, thereby necessitating disproportionate activation under stress, a process that likely accelerates cellular exhaustion and dysfunction rather than reflecting enhanced immune responsiveness, in line with established concepts of immunosenescence and immune exhaustion.

Collectively, our findings indicate that aging reshapes the lymphoid immune system into a dysregulated state, characterized by impaired baseline regulation, exaggerated stress responses, and accelerated B cell differentiation dynamics after sepsis. This is consistent with prior characterizations of immunosenescence as a multisystem dysregulation in the elderly^41,53–55^. This framework helps reconcile why older sepsis patients experience both immune suppression and pathological inflammation and underscores the limitations of “one-size-fits-all” immunotherapies. Age-dependent effects also extended to the inferred drug-target landscape of lymphocytes. Drug2cell analysis suggested that aging alters the transcriptional accessibility of multiple immunoregulatory pathways during sepsis. For example, retinoid X receptor-associated signaling networks exhibited minimal perturbation in young adult mice but showed suppressed baseline activity followed by exaggerated induction in older adults after infection^56,57^. Such findings highlight that precision immunomodulation in sepsis may require age-stratified target selection rather than a universal intervention strategy.

There are several limitations to this study. First, our conclusions are derived exclusively from single-cell transcriptomic profiling and computational inference. While scRNA-seq provides high-resolution insight into cellular composition and gene expression states, validation is needed in future studies by protein-level validation, functional immune assays, or in vivo interventional studies. This study focuses on describing age-dependent transcriptional remodeling rather than definitive functional immune outcomes. Secondly, this study utilized a preclinical murine model of sepsis. While murine models allow mechanistic interrogation not feasible in humans, caution must be taken when translating our findings to human sepsis. Third, all animals were euthanized at a single time point (at day 7 after CLP + DCS) after sepsis induction, a late time point that likely captures post-acute immune remodeling rather than the earliest inflammatory phase of sepsis. Sepsis is a highly dynamic process, and immune responses evolve over time. Our study, therefore, captures a defined snapshot of lymphoid remodeling rather than its full temporal trajectory. This snapshot also excludes mice that died before day 7. Because pre-harvest mortality was higher in older adult septic mice, the surviving cohort represents the more resilient samples of the aged population, and our findings might not capture the full magnitude of age-driven lymphoid dysregulation. Fourth, female mice were analyzed without estrous cycle synchronization. Cyclic fluctuations in sex hormones can modulate lymphocyte function and likely add within-female variance. Fifth, the purpose of Drug2cell in this study was to illustrate age-dependent differences in predicted target landscapes rather than to nominate specific agents for clinical translation.

## Methods

### Animal models.

All animal procedures adhered to protocols approved by the Institutional Animal Care and Use Committee (IACUC) at the University of Florida (UF). C57BL/6J (B6) mice were purchased from Jackson Laboratories (Bar Harbor, ME) and housed at the UF Animal Care Services facility under standard conditions. Animals were provided *ad libitum* feeding on a 12-hour light/dark cycle. Mice were co-housed for a minimum of two weeks prior to experimentation to allow their microbiome to equilibrate due to their coprophagic nature^58^.

### Preclinical rodent sepsis model.

The study cohort consisted of young adult (3–4 months) and older adult (18–24 months), male and female C57BL/6 (B6) mice, stratified into naive control and septic experimental groups. Naive controls were age- and sex-matched, untouched mice that received no surgery or chronic stress. Female mice were included without synchronization of the estrous cycle. Sepsis was induced via cecal ligation and puncture combined with daily chronic stress for seven days (CLP + DCS 7d), as previously described^34,35^. At day seven post-induction, surviving animals were euthanized and spleens were harvested for downstream analysis. Septic mice that died prior to day 7 were excluded from single-cell profiling; the samples reported in **Supplementary Table 1** represent the final sequenced and analyzed cohort. This preclinical murine model follows MQTiPPS recommendations^59^.

### ScRNA-seq and data processing

#### 1. Sample preparation and sequencing.

Splenocytes were harvested, and cell counts were quantified by the Cellometer^™^ Auto 2000 Cell Viability Counter (Nexcelom Bioscience, Lawrence, MA). Cell suspensions were adjusted to a concentration of one million cells per milliliter, requiring a minimum of 85% viability, for scRNA-seq using the Chromium X system (10X Genomics, Pleasonton, CA). Libraries were prepared with the 10X’s Chromium Next GEM Single Cell 5’ v2 kit, targeting 10,000 cells per animal. To ensure quality during library preparation, RNA integrity and dsDNA insert were assessed by the Tapestation 4200 (Agilent, Santa Clara, CA) and Qubit 4 (Invitrogen, Carlsbad, CA). Final concentration of the library pool was quantified with the NEBNext Library Quant Kit (New England Biolabs, Ipswich, MA). Sequencing was conducted on the Illumina Novaseq X plus Platform (Illumina, San Diego, CA) at a minimum depth of 20,000 read pairs per cell.

#### 2. Data preprocessing

Raw sequencing data was demultiplexed using Illumina BCL Convert to generate FASTQ files. Alignment and quantification were performed using 10x Genomics Cell Ranger (v7.0.1)^60^. Reads were mapped to the *Mus musculus* reference transcriptome (mm10; 10x Genomics reference version 2020-A) using the “include-introns” mode, with an expected cell count of 10,000. Gene expression was quantified at the single-cell level, generating unique molecular identifier (UMI) count matrices for downstream analysis.

#### 3. Quality control, integration and clustering.

The feature-barcode matrices were imported into R (v4.3.2) for analysis using the Seurat package (v5.0.1)^61^. Quality control (QC) was performed to filter out low-quality cells (cells with > 10% mitochondrial transcripts content or < 200 detected genes) and potential doublets (cells > 6000 detected genes). Following filtering, gene expression data were log-normalized and scaled. The top 2,000 highly variable genes were selected for dimensionality reduction via Principal Component Analysis (PCA). To correct for batch effects, data integration was performed using Harmony (v1.2.0)^62^, adjusting the top 50 principal components (PCs) to align sample embeddings and reduce batch-specific variation. A Shared Nearest Neighbor (SNN) graph was constructed from 30 nearest neighbors based on the top 30 Harmony-adjusted PCs. Cell clusters were identified by unsupervised clustering with the Louvain algorithm^63^ and visualized in Uniform Manifold Approximation and Projection (UMAP)^64^ embeddings computed from the same Harmony-adjusted PCs. The data including cell clustering patterns, gene expression and RNA velocity were visualized in the first two UMAP dimensions.

#### 4. Cell type annotation.

Clusters were manually annotated by assigning to biological cell types based on the expression of canonical marker genes, following our previous study^36^. Major lymphocyte subsets, including T cells, B cells, natural killer cells, and plasma cells, were first resolved, alongside broader myeloid and dendritic compartments. T cells were further classified into CD4^+^, CD8^+^, and regulatory T (Treg) cells. The subtypes of myeloid cell were identified by re-clustering within the myeloid cells. Erythrocytes and platelets were identified and excluded from downstream analyses, as they were not target populations and likely represented contaminants during library preparation.

### Bioinformatics and Statistical analyses.

After preprocessing, we performed a comprehensive set of analyses focusing on the lymphocyte compartment.

#### 1. Statistical framework for effect testing.

We constructed a statistical framework to quantify how host factors (i.e., age and sex) modified the transcriptional response to sepsis. In this framework, age, sex, and disease (i.e., sepsis) were modeled as independent variables with potential main effects on lymphocyte transcriptomic profiles. We modeled their interaction (age × sepsis, sex × sepsis) effects to assess whether the effect of sepsis varied depending on age and sex. The general model was specified as:

(1) $$Y\sim \beta_{0}+\beta_{1}age+\beta_{2}sex+\beta_{3}\text{sepsis}+\beta_{4}(age\times \text{sepsis})+\beta_{5}(sex\times \text{sepsis})\#$$

Where *Y* represents the biological outcomes of interest (e.g., gene expression, intercellular communication strength, or velocity scores) with appropriate transformations applied when necessary (e.g., log-link GLM for differential expression analysis). This framework decomposes observed variation into: (1) main effects (*β*_1_, *β*_2_, *β*_3_): capturing the effects of age (older vs. young adult), sex (female vs. male), and disease status (sepsis vs. naive), and (2) effect modification terms (*β*_4_, *β*_5_): quantifying how the sepsis effect is altered by age and sex separately. Reference levels were set for young adults, males, and naive. This unified modeling framework was applied across all analyses in this study.

The statistical analysis was performed using R (v4.3.2). Multiple testing was controlled by the Benjamini-Hochberg procedure, with a false discovery rate (FDR) < 0.05 considered statistically significant.

#### 2. Cell type composition differential analysis.

Differences in cell type composition between groups were evaluated by comparing the proportions (relative abundance) of major immune cell populations within all cell types or only the lymphocyte compartment in each sample. The statistical model described in Eq. 1 was used for statistical testing the effect of host factors on cell type relative abundance.

#### 3. Differential expression analysis.

Differential expression analysis was performed on pseudobulk transcriptome profiles generated for each cell type per sample, which has been shown to outperform single-cell-level analysis^65^. For each sample, gene counts were aggregated by cell types (B cells, plasma cells, CD4^+^ T cells, CD8^+^ T cells, Treg cells, and NK cells) using the AggregateExpression function in Seurat^61^. Differential expression analysis was then conducted for each cell type to the pseudobulk count data independently using DESeq2 (v1.40.2)^66^, applying the statistical model described in Eq. 1.

#### 4. Gene set enrichment analysis.

Gene Set Enrichment Analysis (GSEA) was performed to characterize enriched biological pathways represented by the differentially expressed genes. The analysis was conducted using the clusterProfiler package (v4.10.1)^67^ with the Gene Ontology Biological Process database (GOBP)^68^ from Molecular Signatures Database (MSigDB)^69^ as the reference database. For each lymphocyte subtype, genes were first ranked by the signed Wald statistic derived from the DESeq2 analysis for each model term (main effects and interactions, as modeled in Eq. 1). Enrichment with FDR < 0.05 was considered statistically significant. The direction of regulation was inferred from the sign of the Normalized Enrichment Score (NES), where positive values indicated pathway activation and negative values indicated suppression.

#### 5. Intercellular communication analysis.

To infer and compare intercellular communication networks, we utilized the R package CellChat (v2.2.0)^70^. Initially, a CellChat object was created for each biological sample independently using the CellChatDB.mouse ligand-receptor database as the reference. Within each sample, communication probabilities were inferred using the truncated mean (triMean) method and subsequently aggregated at the signaling pathway level. Our subsequent analyses focused specifically on interactions among T cells (CD4^+^ T, CD8^+^ T, Treg) and antigen-presenting cells (APCs), which included B cells, plasma cells, macrophages, conventional type 1 dendritic cell (cDC1), conventional type 2 dendritic cell (cDC2), and plasmacytoid dendritic cell (pDC). To quantify how experimental variables affected overall cell-cell communication, we first calculated the aggregated communication strength. This metric represents the total communication strength summed across all ligand-receptor pairs (L-R pairs) for a specific source cell type to target cell type communication. Then, each unique source-to-target pair was further fit by the statistical model (Eq. 1) to assess the effect of experimental variables on specific intercellular communication pathways.

#### 6. RNA velocity analysis.

To infer cellular dynamics and trajectories, RNA velocity analysis was performed based on the spliced and unspliced mRNA counts quantified by velocyto (v0.17.17)^71^. The RNA velocity inference was conducted in Python (v3.10.17) using scVelo package (v0.3.3)^72^. Briefly, data underwent cell-wise normalization, and selection of the top 2,000 highly variable genes. The neighborhood graph (k = 30 neighbors) and moments were computed using the Harmony-corrected PCA representation. Velocities were estimated using the stochastic model implemented in scVelo, and a velocity graph was constructed to learn cellular transition probabilities. Single-cell velocity score, a proxy for the magnitude of cell state change and cell differentiation speed, was calculated for each cell. This metric was then used as the dependent variable in the statistical modeling framework (Eq. 1), for assessing the main and interaction effect of age, sex and disease on RNA velocity strength. Specifically, we modeled the log_10_(velocity score) using a linear mixed model (LMM) with experimental variables as fixed effects and a random intercept for each sample to account for correlation structure.

#### 7. Drug-cell targeting analysis.

To map the landscape of potential pharmacological targets at single-cell level, we performed drug-cell targeting analysis using the Drug2cell (v0.1.1)^37^. Drug-target interactions were referenced from the orthologous mapped ChEMBL database^73^, with human target genes converted to mouse homologs using Ensembl BioMart^74^. Drug2cell resulted in a cell-by-drug score matrix representing the potential drug responsiveness based on transcriptomic signatures. The top drug candidates were identified empirically with mean drug score > 0.01. To statistically evaluate differential activity of drug targeting, we adopted a pseudobulk approach similar to differential expression analysis. Single-cell drug scores were averaged at sample level for each of six key lymphocyte populations (B cells, plasma cells, CD4^+^ T, CD8^+^ T, Treg cells, and NK cells). These sample-level means were then used as the dependent variable in the statistical model (Eq. 1) to identify therapeutic targets significantly modulated by sepsis, age, or sex.

## Supplementary Material

Supplementary Files

This is a list of supplementary files associated with this preprint. Click to download.

- Suppmaterials0518.pdf

## Figures and Tables

**Table 1:** Number of differentially expressed genes (DEGs) in lymphocyte subsets. The table displays the count of significant DEGs (adjusted p < 0.05) for each cell type. Direction of differential expression (log2 fold change) is relative to specific reference groups: naive (for disease), young adult (for age), and male (for sex). “up” and “down” indicate significantly upregulated (positive LFC) or downregulated (negative LFC) genes in the test condition. For interaction terms, “positive” denotes genes more upregulated (or less downregulated) in the combined condition, while “negative” indicates the inverse.

	Sepsis		Age		Sex		Age × sepsis		Sex × sepsis	
	Up	Down	Up	Down	Up	Down	Positive	Negative	Positive	Negative
**B**	2,755	3,111	4,501	4,008	108	73	1,312	642	0	0
**Plasma**	1,288	1,692	2,876	3,100	13	39	141	72	10	1
**CD4^+^ T**	550	226	4,039	3,402	12	8	889	454	0	0
**CD8^+^ T**	629	610	4,955	4,504	16	8	1,980	630	0	0
**Treg**	511	562	4,637	4,125	82	24	1,796	569	0	0
**NK**	1,321	1,225	4,202	3,819	12	7	1,646	631	0	0

**Table 2:** Transcriptional overlap between sepsis-associated and age-associated genes. The table displays the count of genes significantly differentially expressed (adjusted p < 0.05) in both sepsis (vs. naive) and age (older adult vs. young adult) comparisons. Columns categorize overlaps by directionality: “↑” indicates upregulation and “↓” indicates downregulation relative to reference groups. The middle columns (↑ sepsis, ↓ age and ↓ sepsis, ↑ age) represent the “Inverse” relationship, identifying genes where the sepsis response opposes the aging effect. P-values (Fisher’s exact test) indicate the statistical significance of the association between sepsis-driven and age-driven transcriptional changes.

Cell type	↑ Sepsis, ↑ Age	↑ Sepsis, ↓ Age	↓ Sepsis, ↑ Age	↓ Sepsis, ↓ Age	P value
**B**	148	1,615	2,071	83	< 0.001
**Plasma**	144	470	620	156	< 0.001
**CD4^+^ T**	216	62	141	23	0.03
**CD8^+^ T**	149	123	446	38	< 0.001
**Treg**	115	184	433	27	< 0.001
**NK**	142	624	861	81	< 0.001

**Table 3:** Summary of age-dependent modulation of lymphocyte responses to sepsis.

Domain	Sepsis effect	Age effect	Age × sepsis interaction
**Lymphocyte composition**	Global lymphocyte decrease with plasma cell expansion.	Reduced T/NK cells and expanded B cells at baseline.	Attenuated T/NK decrease but enhanced B→ plasma shift in older adults during sepsis.
**Pathway activity**	Upregulation of metabolic pathways, downregulation of immune and proliferative pathways.	Downregulation of metabolic pathways, upregulation of immune and proliferative pathways at baseline.	Exaggerated sepsis-driven metabolic pathways upregulation, and immune/proliferative downregulation in older adult mice.
**APC-to-T cell communication**	Downregulation of APC→CD4^+^ T cells signaling.	Global downregulation of APC-to-T cell communication at baseline.	Relative preservation or enhanced APC-to-T cell signaling in older adult mice during sepsis (CD4^+^ T: IL16, ICOS, ICAM, MHC-II; CD8^+^ T: ICOS, CD86).
**Cell differentiation**	Accelerated B cell differentiation toward plasma cells.	Reduced B cell and plasma cell differentiation at baseline.	Exaggerated sepsis-driven B-to-plasma cell differentiation in older adult mice.
**Drug targeting potential**	Global remodeling of inferred drug-targeting landscape.	Age-associated shift in inferred drug-targeting landscape at baseline.	Age-dependent reprogramming of the sepsis-responsive drug-targeting landscape. Several molecules exhibited age-dependent increase of inferred drug targeting potential.

## Data Availability

The single-cell RNA sequencing datasets generated and analyzed during the current study are publicly available in the NCBI Gene Expression Omnibus (GEO) repository under accession number GSE311155 (https://www.ncbi.nlm.nih.gov/geo/query/acc.cgi?acc=GSE311155). The associated raw sequencing reads are deposited in the NCBI Sequence Read Archive (SRA) under BioProject PRJNA1368753 (https://www.ncbi.nlm.nih.gov/bioproject/PRJNA1368753). Any additional information required to reanalyze the data reported in this paper is available from the corresponding author upon reasonable request.
